# Two novel, putative mechanisms of action for citalopram-induced platelet inhibition

**DOI:** 10.1038/s41598-018-34389-5

**Published:** 2018-11-12

**Authors:** Harvey G. Roweth, Aaron A. Cook, Masaaki Moroi, Arkadiusz M. Bonna, Stephanie M. Jung, Wolfgang Bergmeier, Stewart O. Sage, Gavin E. Jarvis

**Affiliations:** 1 Department of Physiology, Development and Neuroscience, University of Cambridge, Cambridge, UK; 2 Department of Biochemistry and Biophysics, University of North Carolina at Chapel Hill, Chapel Hill, USA; 3 Department of Biochemistry, University of Cambridge, Cambridge, UK

## Abstract

Citalopram, a selective serotonin reuptake inhibitor (SSRI), inhibits platelet function *in vitro*. We have previously shown that this action is independent of citalopram’s ability to block serotonin uptake by the serotonin transporter and must therefore be mediated via distinct pharmacological mechanisms. We now report evidence for two novel and putative mechanisms of citalopram-induced platelet inhibition. Firstly, in platelets, citalopram blocked U46619-induced Rap1 activation and subsequent platelet aggregation, but failed to inhibit U46619-induced increases in cytosolic Ca^2+^. Similarly, in neutrophils, citalopram inhibited Rap1 activation and downstream functions but failed to block PAF-induced Ca^2+^ mobilisation. In a cell-free system, citalopram also reduced CalDAG-GEFI-mediated nucleotide exchange on Rap1B. Secondly, the binding of anti-GPVI antibodies to resting platelets was inhibited by citalopram. Furthermore, citalopram-induced inhibition of GPVI-mediated platelet aggregation was instantaneous, reversible and displayed competitive characteristics, suggesting that these effects were not caused by a reduction in GPVI surface expression, but by simple competitive binding. In conclusion, we propose two novel, putative and distinct inhibitory mechanisms of action for citalopram: (1) inhibition of CalDAG-GEFI/Rap1 signalling, and (2) competitive antagonism of GPVI in platelets. These findings may aid in the development of novel inhibitors of CalDAG-GEFI/Rap1-dependent nucleotide exchange and novel GPVI antagonists.

## Introduction

Citalopram, a selective serotonin reuptake inhibitor (SSRI), is widely used as an antidepressant^1^. Its primary pharmacological target is the serotonin transporter (SERT)^2,3^ inhibition of which prevents cellular uptake of serotonin (5-hydroxytryptamine, 5-HT)^4^. SSRIs are widely believed to exert psychiatric benefit by inhibiting SERT and modifying serotonergic neurotransmission in the central nervous system^5^. SERT is also found on non-neuronal cells, including platelets, which store 5-HT in dense granules that resemble neurotransmitter vesicles^6,7^. Citalopram not only inhibits platelet SERT, but also platelet aggregation, adhesion, thromboxane A_2_ (TxA_2_) synthesis and dense granule release^8–12^. However, this functional inhibition is not caused by blockade of 5-HT uptake and must therefore be mediated by distinct pharmacological mechanisms of action^10^.

Citalopram inhibits platelet aggregation induced by both collagen and the TxA_2_ mimetic, U46619, countering the claim that it is a specific inhibitor of collagen-induced platelet activation^9^. Nevertheless, citalopram is a more potent inhibitor of collagen^10^, which activates platelets predominantly via glycoprotein VI (GPVI), than U46619, a thromboxane prostanoid (TP) receptor agonist, suggesting differential mechanisms of action. Reduced phosphorylation of signalling proteins in the GPVI pathway^10^ points to GPVI as a possible site of action for citalopram, which could act as a classical competitive antagonist or allosteric inhibitor. For example, citalopram could disrupt GPVI dimers that mediate collagen binding and platelet activation^13^. However, GPVI inhibition would not account for citalopram’s effect on U46619-induced responses.

Both GPVI and TP receptor activation raise cytosolic calcium concentrations ([Ca^2+^]_cyt_), a shared signalling pathway for collagen, TxA_2_, and many other platelet agonists. Although there are numerous reports of citalopram inhibiting platelet aggregation *in vitro*^8–12^ few have directly measured its effect on Ca^2+^ signalling. Tseng *et al*.^14^ reported that citalopram did not inhibit ADP-induced Ca^2+^ signalling, suggesting some specificity in the action of citalopram, perhaps downstream of increased [Ca^2+^]_cyt_. Elevated [Ca^2+^]_cyt_ upregulates GTP binding to the small GTPase Rap1, a process catalysed by the calcium and diacylglycerol guanine nucleotide exchange factor-1 (CalDAG-GEFI, also known as RAS guanyl-releasing protein 2)^15–17^. Rap1-GTP mediates the transition of integrin α_IIb_β_3_ (also known as glycoprotein (GP) IIb/IIIa) to a high-affinity state, thereby facilitating platelet aggregation through fibrinogen crosslinks^18,19^.

Therefore, our search for novel mechanisms for citalopram focussed on GPVI and Ca^2+^ signalling. We utilised the selective GPVI agonist, collagen-related peptide (CRP), and also investigated Ca^2+^ signalling in neutrophils, which share similar Ca^2+^-dependent mechanisms of activation^20,21^ and are also inhibited by SSRIs *in vitro*^22^. Our data reveal two novel, putative inhibitory mechanisms of action for citalopram.

## Results

### MECHANISM 1: Inhibition of CalDAG-GEFI-dependent Rap1 nucleotide exchange

#### Differential inhibition of GPVI-dependent and U46619-induced calcium mobilisation

Citalopram inhibits both collagen- and U46619-induced platelet aggregation^10^. Since aggregation is dependent on Ca^2+^ signalling, the effect of citalopram on agonist-induced increases in [Ca^2+^]_cyt_ was investigated. Cross-linked CRP (CRPXL), a selective agonist for GPVI, was used instead of collagen to generate exclusively GPVI-dependent responses. [Ca^2+^]_cyt_ was monitored in platelets pre-treated for approximately 5 min with citalopram (0, 10, 20, 50, 100 & 200 µM), before stimulation with either CRPXL (0.5 µg mL^−1^) or U46619 (0.2 µM). These just sub-maximal concentrations of CRPXL and U46619 were selected based on prior pilot experiments (Supplementary Fig. S1).

**Figure 1:**
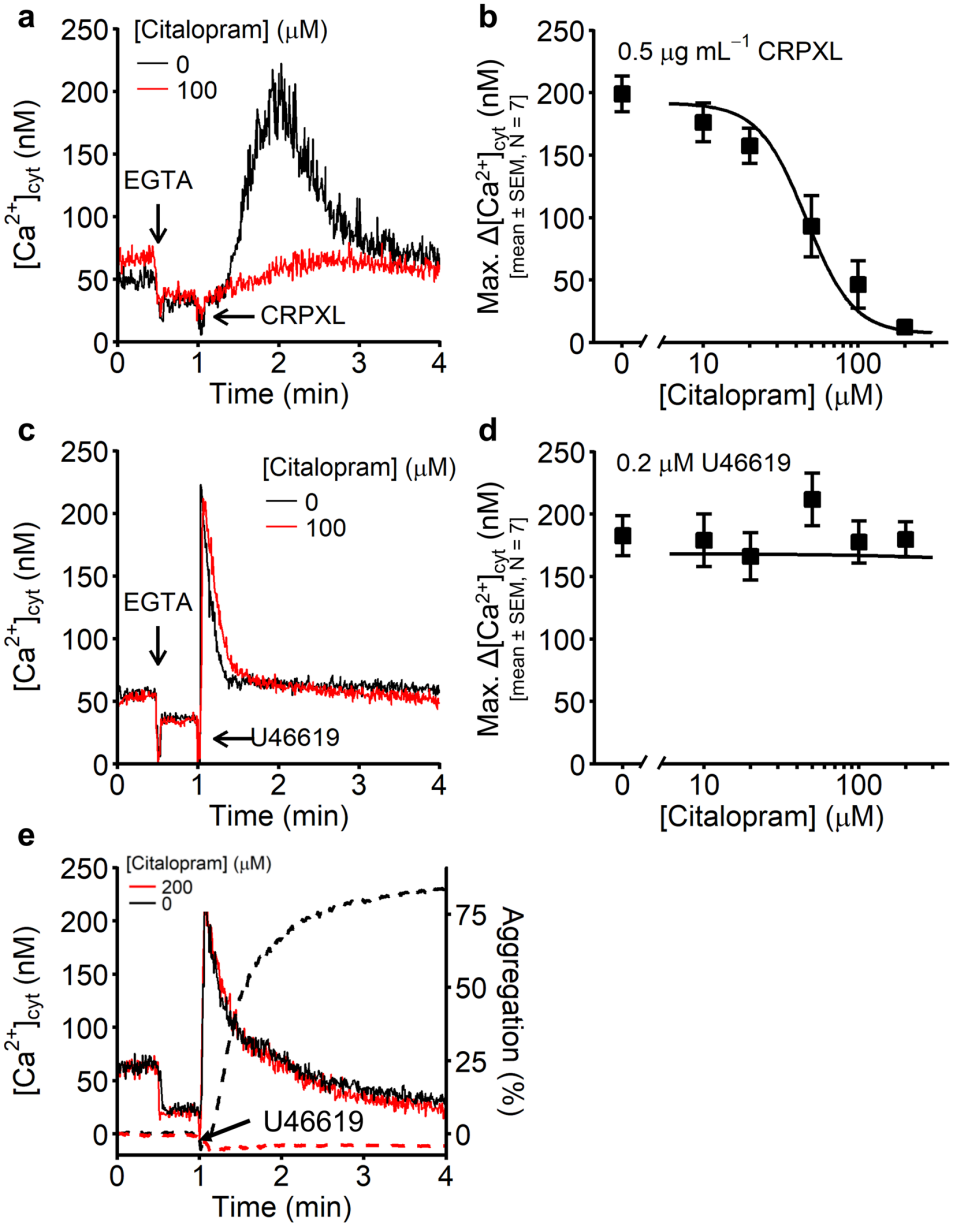
Calcium release from intracellular stores in either untreated or citalopram-treated platelets. Example traces are shown for both (**a**) cross-linked collagen-related peptide (CRPXL)-stimulated (0.5 µg mL^−1^) and (**c**) U46619-stimulated (0.2 µM) platelets, either untreated or pre-treated with citalopram (100 µM) for approximately 5 min. (**b**,**d**) After the addition of agonist, the cytosolic concentration of Ca^2+^ ([Ca^2+^]_cyt_) in platelets pre-incubated with citalopram (0, 10, 20, 50, 100 & 200 µM) was recorded for 3 min. The maximum increase in [Ca^2+^]_cyt_ following agonist addition (Max. Δ[Ca^2+^]_cyt_) was used to generate concentration-response curves, using the four-parameter logistic (4PL) model (N = 7 blood donors). (**e**) In a separate experiment, using platelets from the same blood donor on the same experimental day, [Ca^2+^]_cyt_ (solid lines) and aggregation (dashed line) were separately recorded following pre-incubation with or without citalopram (200 µM) and stimulation with U46619 (0.2 µM) (N = 1 blood donor).

CRPXL-induced increases in [Ca^2+^]_cyt_ were abolished by citalopram (Fig. 1a,b). The inhibitory potency of citalopram (*pIC*_50_ = 4.34 ± 0.09 (N = 7 blood donors)) matched its potency for inhibiting collagen-induced aggregation (*pIC*_50_ = 4.31 ± 0.21^10^). This is also consistent with our previous observations that citalopram inhibited tyrosine phosphorylation signalling downstream of GPVI^10^. Surprisingly, and in stark contrast, citalopram had no discernible effect on U46619-induced increases in [Ca^2+^]_cyt_ (Fig. 1c,d) (N = 7 blood donors).

To confirm that Fura-2-loading had no functional effect on platelets, on one occasion, U46619-induced aggregation was measured in the same preparation of Fura-2-loaded platelets used for Ca^2+^ measurements. Fura-2-loaded platelets aggregated normally in response to 0.2 µM U46619 (Fig. 1e) and citalopram (200 µM) inhibited this response, as expected. However, as reported above, the increase in [Ca^2+^]_cyt_ was unaffected by citalopram.

Thus, in citalopram-treated platelets, U46619 induced a normal Ca^2+^ response, but no aggregation, suggesting that citalopram inhibits aggregation at a point downstream of Ca^2+^ mobilisation. This conclusion was supported by the observation that citalopram also inhibited platelet aggregation induced by the Ca^2+^ ionophore, ionomycin (0.5 µM), in a concentration-dependent manner: *pIC*_50_ = 3.98 ± 0.09 (N = 4 blood donors) (Supplementary Fig. S2).

#### Citalopram inhibits Rap1 activation in platelets

U46619-induced platelet activation was inhibited by citalopram, despite preserved Ca^2+^ store release (Fig. 1). We therefore aimed to identify where citalopram exerts its inhibitory effects downstream of Ca^2+^ release.

**Figure 2:**
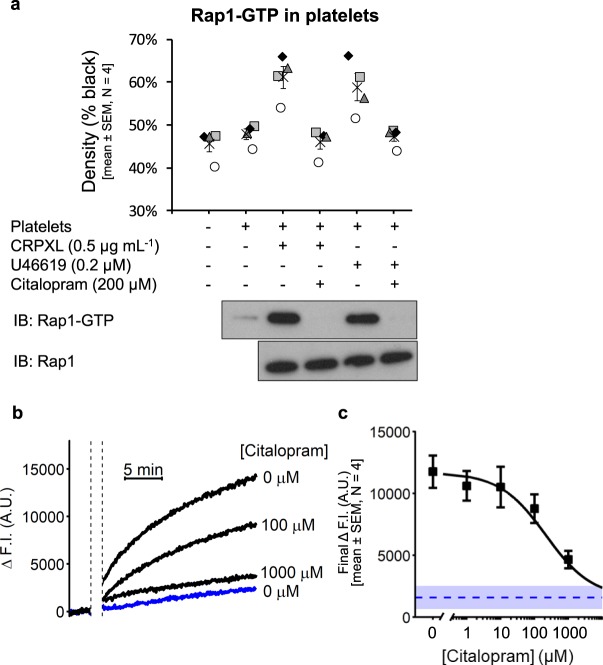
Citalopram inhibits Rap1-GTP formation in platelets. **(a)** Platelets were pre-treated with (+) or without (−) citalopram (200 µM) for approximately 5 min, before stimulation for 1 min with either cross-linked collagen-related peptide (CRPXL, 0.5 µg mL^−1^) or U46619 (0.2 µM). Rap1-GTP was isolated from unstimulated and agonist-stimulated platelets and quantified using densitometry following SDS-PAGE and Western blotting. Total Rap1 levels were also measured. (N = 4 blood donors. Different donors are indicated by different symbols and the mean by ×). (**b**) Example traces from a BODIPY-FL-GDP fluorescence-based assay, which was used to monitor the nucleotide exchange activity of Rap1B. CalDAG-GEFI was pre-incubated with citalopram (0, 100 & 1000 µM) for approximately 5 min before its addition to wells (white segment, black dashed lines) containing BODIPY-FL-GDP and Rap1B. The blue trace indicates fluorescence in the absence of CalDAG-GEFI. (**c**) Following CalDAG-GEFI pre-incubations with citalopram (0, 1, 10, 100 & 1000 µM), the increase in fluorescence intensity 20 min after the addition of CalDAG-GEFI (Final ΔF.I.) was used to create concentration-response curves. The blue dashed line (mean), and blue area (±SEM) show the Final ΔF.I. in the absence of CalDAG-GEFI, which was used to constrain the *Max* parameter of the four-parameter logistic (4PL) model (N = 4 experiments).

Increased [Ca^2+^]_cyt_ causes CalDAG-GEFI-mediated Rap1 activation and downstream platelet aggregation^15,17^. Experiments were conducted to determine if citalopram inhibits U46619-induced platelet aggregation by preventing Rap1 activation. The effect of citalopram (200 µM) on Rap1 activation induced by CRPXL (0.5 µg mL^−1^) or U46619 (0.2 µM) was investigated. Activated Rap1 (Rap1-GTP) was isolated and quantified by Western blot analysis. The results (Fig. 2a) clearly show that citalopram inhibited both CRPXL- and U46619-induced Rap1-GTP formation (*P* = 7.5 × 10^−9^, F = 83.4, as determined by regression analysis (Supplementary Analysis 1)). Thus, Rap1 activation is blocked by concentrations of citalopram that inhibit U46619-induced platelet aggregation, but have no effect on Ca^2+^ release from intracellular stores.

Further experiments were conducted to investigate whether citalopram suppresses CalDAG-GEFI-mediated nucleotide exchange of isolated Rap1, using purified recombinant CalDAG-GEFI and Rap1B, the predominant Rap1 isotype in platelets^17,23^. Nucleotide exchange was monitored by detecting fluorescent BODIPY-FL-conjugated GDP as described in the Methods. Citalopram inhibited CalDAG-GEFI-mediated BODIPY-FL-GDP exchange onto Rap1B in a concentration-dependent manner (Fig. 2b,c). Peak increases in fluorescence intensity (ΔF.I.) were fitted to the four-parameter logistic (4PL) model, with the *Max* parameter constrained to the basal response, indicated by the ΔF.I. observed when no CalDAG-GEFI was added. The *pIC*_50_ value was 3.67 ± 0.32 (N = 4 experiments).

#### Citalopram inhibits Rap1 activation in neutrophils

The results above indicate that citalopram inhibits CalDAG-GEFI-dependent Rap1B nucleotide exchange and imply that other cells expressing CalDAG-GEFI/Rap1 would also be inhibited by citalopram. Similarly to platelets, neutrophils express both CalDAG-GEFI and Rap1 (the predominant form is also Rap1B^24^), which mediate agonist and Ca^2+^-dependent activation^21,25^. Signalling and functional studies were therefore conducted on isolated neutrophils to determine the effects of citalopram treatment.

**Figure 3:**
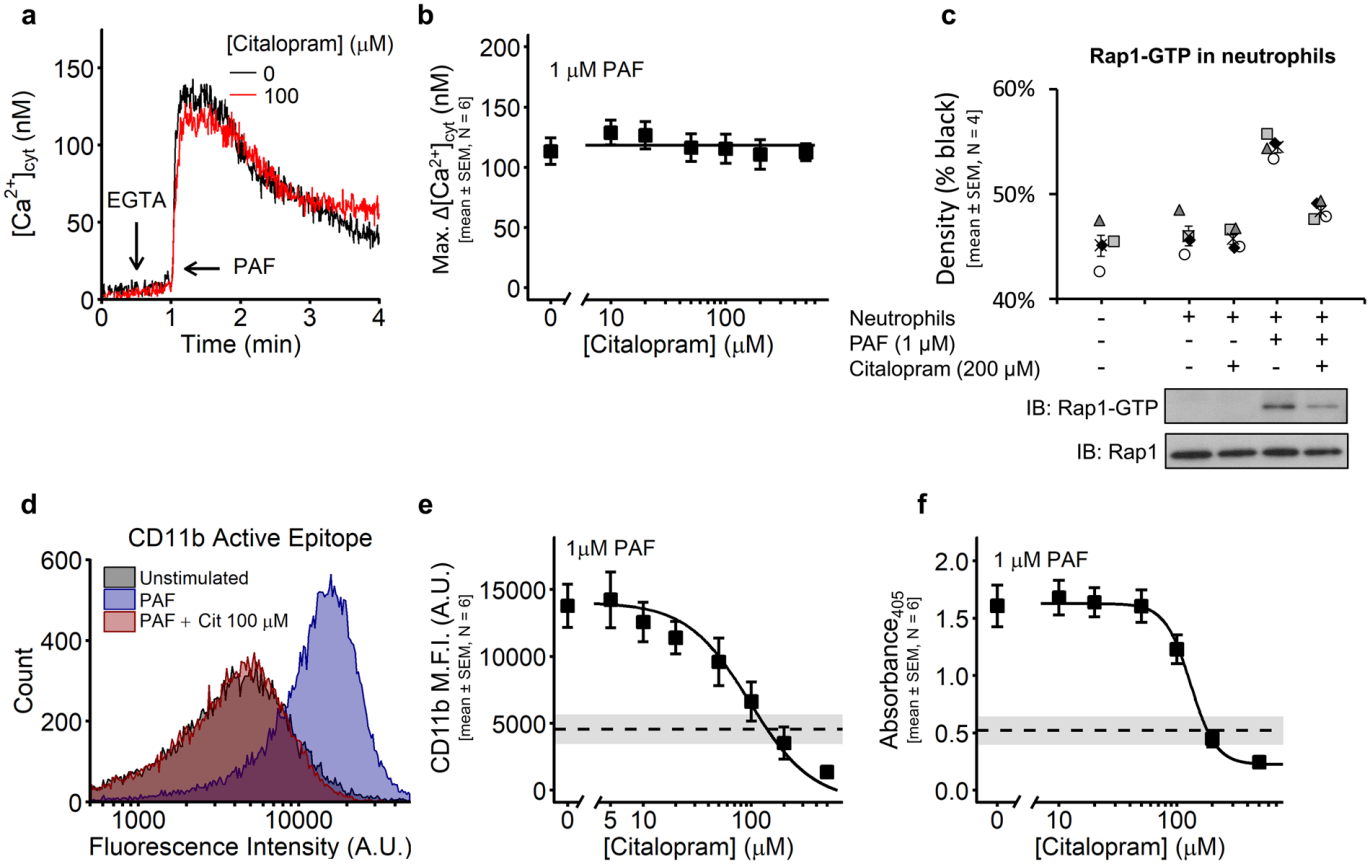
Citalopram inhibits neutrophil functions, despite preserved calcium store release. (**a**,**b**) Calcium (Ca^2+^) store release was monitored in Fura-2-loaded neutrophils, stimulated with 1 µM platelet-activating factor (PAF). (**a**) Example traces demonstrate PAF-induced increases in the cytosolic concentration of Ca^2+^ ([Ca^2+^]_cyt_). (**b**) Neutrophils were pre-incubated for approximately 5 min with citalopram (0, 10, 20, 50, 100, 200 & 500 µM) prior to the addition of PAF. The maximum increase in [Ca^2+^]_cyt_ following PAF addition (Max. Δ[Ca^2+^]_cyt_) was recorded (N = 6 blood donors). (**c**) Neutrophils were pre-incubated with (+) or without (−) citalopram (200 µM) for approximately 5 min, followed by either no stimulation (−) or stimulation (+) with 1 µM PAF for 1 min. Rap1-GTP was isolated and quantified using densitometry following SDS-PAGE and Western blotting (N = 4 blood donors. Different donors are indicated by different symbols and the mean by ×). (**d**) Representative histograms, measuring antibody binding to the active epitope of α_M_ (CD11b) in either unstimulated or PAF-stimulated neutrophils (citalopram = 0 or 100 µM, PAF = 1 µM). (**e**,**f**) A range of citalopram concentrations (0, 5, 10, 20, 50, 100, 200 & 500 µM) were used to create concentration-response curves for the effects of citalopram on **(e)** monoclonal antibody binding to CD11b and (**f**) neutrophil adhesion to fibrinogen (N = 6 blood donors). Dashed line (mean) and grey area (±SEM) indicate (**e**) α_M_β_2_ activation or (**f**) fibrinogen-binding in unstimulated neutrophils.

Platelet-activating factor (PAF) is a potent activator of neutrophils. PAF-induced (1 µM) increases in the [Ca^2+^]_cyt_ were measured following a 5 min citalopram pre-treatment (0, 10, 20, 50, 100, 200 & 500 µM). As with platelets, citalopram did not affect PAF-induced increases in [Ca^2+^]_cyt_ in neutrophils (N = 6 blood donors) (Fig. 3a,b), whereas PAF-induced (1 µM) Rap1 activation was inhibited by citalopram (200 µM) (Fig. 3c). (*P* = 5.9 × 10^−6^, F = 38.7, as determined by regression analysis (Supplementary Analysis 2)).

#### Citalopram inhibits neutrophil function

Rap1 regulates the transition of α_M_β_2_ integrin (Mac-1, CD11b/18) to a high-affinity binding state in macrophages^26^. α_M_β_2_ is a cell surface adhesion receptor for fibrinogen^27^ and CalDAG-GEFI-deficient neutrophils stimulated with PAF show impaired α_M_β_2_-dependent adhesion to fibrinogen^21^.

Experiments were performed to determine if citalopram could inhibit activation of neutrophil integrin α_M_β_2_. Neutrophils were pre-incubated with citalopram (0, 5, 10, 20, 50, 100, 200 & 500 µM) for approximately 5 min, followed by PAF stimulation (1 µM). Representative flow cytometry histograms show that citalopram inhibited PAF-induced integrin α_M_β_2_ activation (Fig. 3d). Citalopram inhibited sample median fluorescence intensity (M.F.I.) in a concentration-dependent manner (Fig. 3e: *pIC*_50_ = 4.02 ± 0.15 (N = 6 blood donors)).

The adhesion of PAF-stimulated (1 µM) neutrophils to fibrinogen under static conditions was also investigated to determine if impaired integrin α_M_β_2_ activation resulted in a reduction in cell adhesion. As expected, citalopram (0, 10, 20, 50, 100, 200 & 500 µM) inhibited neutrophil adhesion in a concentration-dependent manner (Fig. 3f: *pIC*_50_ = 3.88 ± 0.04 (N = 10 blood donors)).

The membrane integrity of neutrophils was also assessed to check if impaired functional responses in the presence of citalopram were a result of cell cytotoxicity. Citalopram (0, 10, 20, 50, 100, 200 & 500 µM) had no effect on lactate dehydrogenase (LDH) release (Supplementary Fig. S3: N = 5 blood donors).

Inhibition of neutrophil signalling and function by citalopram closely matched that observed in platelets. Therefore, these data support the hypothesis that citalopram inhibits CalDAG-GEFI-dependent Rap1 nucleotide exchange.

### MECHANISM 2: GPVI antagonism

Inhibition of CRPXL-induced Ca^2+^ signalling by citalopram (Fig. 1a,b) is consistent with our previous observations that GPVI-mediated tyrosine phosphorylation of PLCγ2 is inhibited by citalopram^10^. It also suggests that citalopram has a mechanism of action distinct from the inhibition of CalDAG-GEFI-dependent Rap1 nucleotide exchange. Given that citalopram also reduces phosphorylation of FcRγ chain and Src family kinases (SFKs)^10^ we hypothesised that citalopram may have a direct effect on GPVI structure and/or function.

#### Citalopram inhibits the binding of GPVI antibodies

GPVI is expressed on the surface of platelets in both monomeric and dimeric conformations, although the dimeric form is thought to be particularly important in collagen binding and subsequent platelet activation^13,28^. We hypothesised that citalopram may disrupt the dimeric structure of GPVI, thereby preventing collagen- and CRPXL-induced responses. Experiments were conducted using antibodies that selectively detect either dimeric GPVI (204-11 Fab fragments) or total (dimeric and monomeric) GPVI (HY-101) to determine whether citalopram altered GPVI-dimer expression.

**Figure 4:**
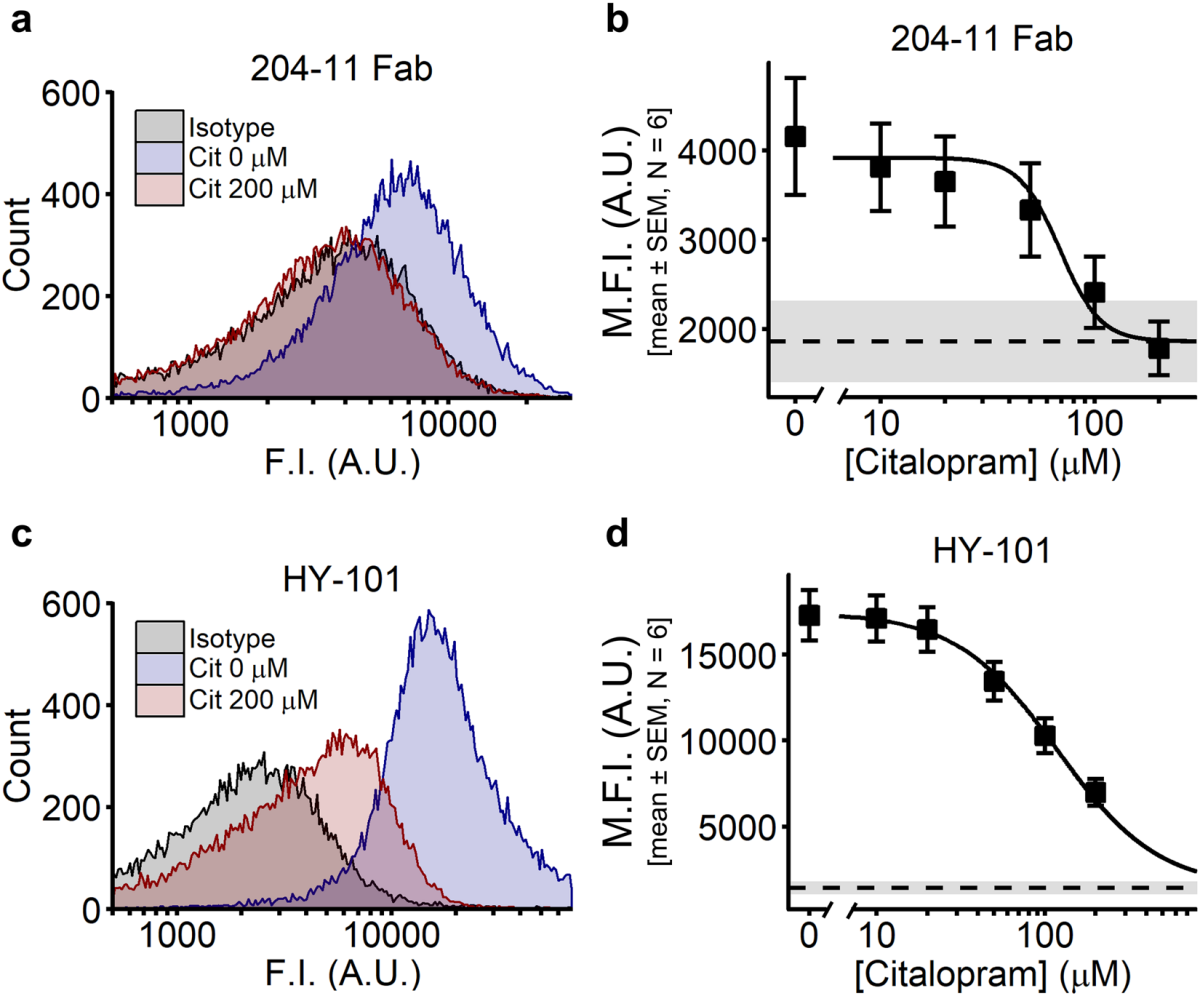
Binding of dimeric anti-glycoprotein VI (GPVI) (204-11 Fab) or total anti-GPVI (HY-101) antibodies to unstimulated platelets. Control Fab or IgG_2a_κ were used as corresponding isotype controls for (**a**,**b**) 204-11 Fab and (**c**,**d**) HY-101, respectively. Example histograms represent the fluorescence intensity (F.I.) of platelets pre-incubated with either a GPVI-specific antibody (blue = 0 µM citalopram, red = 200 µM citalopram), or an isotype control (grey). Sample median F.I. (M.F.I.) was used to generate concentration-response curves, fitted to the four-parameter logistic (4PL) model. Dashed lines (mean) and grey space (±SEM) indicate the M.F.I. of isotype controls, which were used to constrain the *Max* parameter of the 4PL model (N = 6 blood donors).

Citalopram reduced the fluorescence intensity (F.I.) of unstimulated platelets labelled with 204-11 Fab fragments and HY-101 antibodies in a concentration-dependent manner (Fig. 4). M.F.I. from platelet samples were fitted to the 4PL model, with the *Max* parameter constrained to the F.I. of the isotype control (Fig. 4b,d: *pIC*_50_ (204-11) = 4.16 ± 0.03; *pIC*_50_ (HY-101) = 3.93 ± 0.07 (N = 6 blood donors)). These data suggest that citalopram either reduces total GPVI surface expression or blocks the binding of GPVI antibodies to GPVI. The reduction in HY-101 antibody binding suggests that the effect of citalopram is not specific to dimeric GPVI.

#### Citalopram-induced inhibition of GPVI-mediated platelet activation is fully reversible

**Figure 5:**
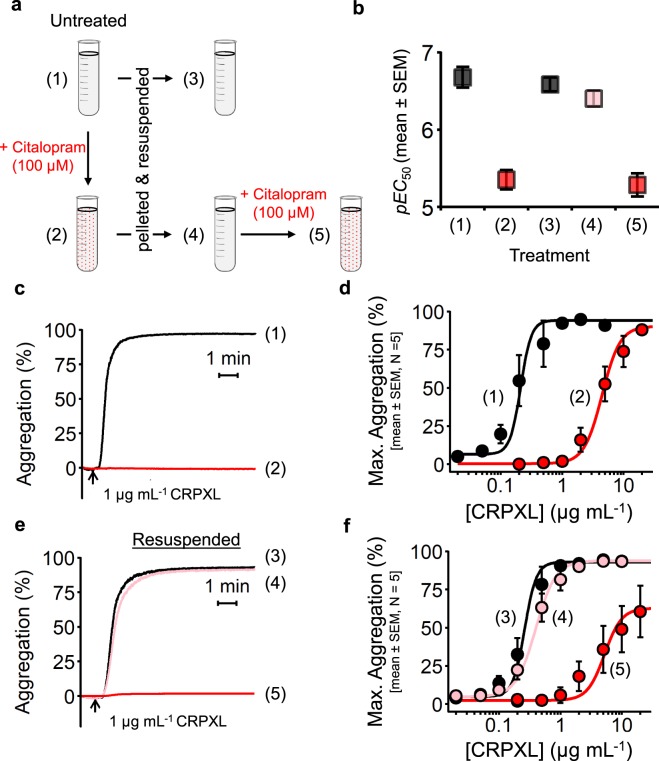
Citalopram inhibition of CRPXL-induced platelet aggregation is reversible. (**a**) Diagram outlining the experimental design to test the reversibility of platelet inhibition by citalopram. Washed platelets were pelleted by centrifugation in the presence of prostaglandin E_1_ (PGE_1_, 1 µM) and resuspended in fresh calcium-free Tyrode’s (CFT). (**b**) *pEC*_50_ values were derived from the concentration-response curves in (**d**,**f**) (N = 5 blood donors). (**c**,**d**) Two stocks of washed platelets were either untreated (1) or treated with 100 µM citalopram (2). Samples were aliquoted to measure CRPXL-induced aggregation. (**e**,**f**) Stocks (1) and (2) were pelleted and resuspended in fresh CFT and left to rest for 1 hour. Platelet aggregation was then measured in untreated resuspended platelets (3), citalopram-treated resuspended platelets (4), and citalopram-treated resuspended platelets with a second citalopram treatment of 100 µM (5).

We next investigated whether the impaired GPVI antibody binding caused by citalopram was due to a functionally irreversible mechanism of action such as receptor shedding or internalisation. Platelets were pre-incubated for approximately 5 min with citalopram (0 & 100 µM), which was subsequently removed by pelleting and resuspending platelets in fresh calcium-free Tyrode’s (CFT) containing no citalopram (Fig. 5a). Platelets were then stimulated with CRPXL, with or without citalopram, under standard aggregometry conditions.

As expected, citalopram (100 µM) inhibited CRPXL-induced platelet aggregation (Fig. 5c,d). Resuspension of citalopram-treated platelets in fresh CFT restored CRPXL-induced aggregation and the resuspended control and citalopram-pre-treated platelets responded similarly to CRPXL. Addition of citalopram (100 µM) to pre-treated, pelleted and resuspended platelets inhibited CRPXL-induced aggregation (Fig. 5e,f).

Concentration-response data for the five different conditions were fitted to the 4PL model and CRPXL *pEC*_50_ values (Fig. 5b) were: (1) untreated = 6.67 ± 0.13; (2) citalopram-treated = 5.35 ± 0.13; (3) untreated and resuspended = 6.58 ± 0.09; (4) citalopram-treated and resuspended = 6.40 ± 0.10; (5) citalopram-treated, resuspended and citalopram-treated = 5.28 ± 0.15; (N = 5 blood donors)). Analysis by 1-way repeated-measures ANOVA (Effect 1 (fixed) = treatment {1,2,3,4,5}; Effect 2 (repeated measure) = donor {N = 5}) indicated a difference between the *pEC*_50_ values of the five treatments (*P* = 1.3 × 10^−9^, F = 63.4, df = 4, 16). A post-hoc Tukey test suggested that there was no difference between the *pEC*_50_ values of untreated platelets and citalopram-treated platelets following resuspension (*P* = 0.59). Similar results were observed for collagen- and U46619-stimulated platelets (Supplementary Figs S4 and S5).

#### Citalopram rapidly inhibits CRPXL-induced platelet aggregation in a competitive manner

As shown above, citalopram inhibited the binding of anti-GPVI antibodies and platelet stimulation by CRPXL. However, this latter effect was fully reversible, suggesting that citalopram may bind reversibly to GPVI, thereby preventing binding of the anti-GPVI antibodies and CRP. Such a mechanism would be rapid in onset and competitive in character. We therefore performed additional experiments to investigate the kinetics of onset of platelet inhibition by citalopram, and whether it exhibited a competitive or non-competitive pattern of inhibition.

**Figure 6:**
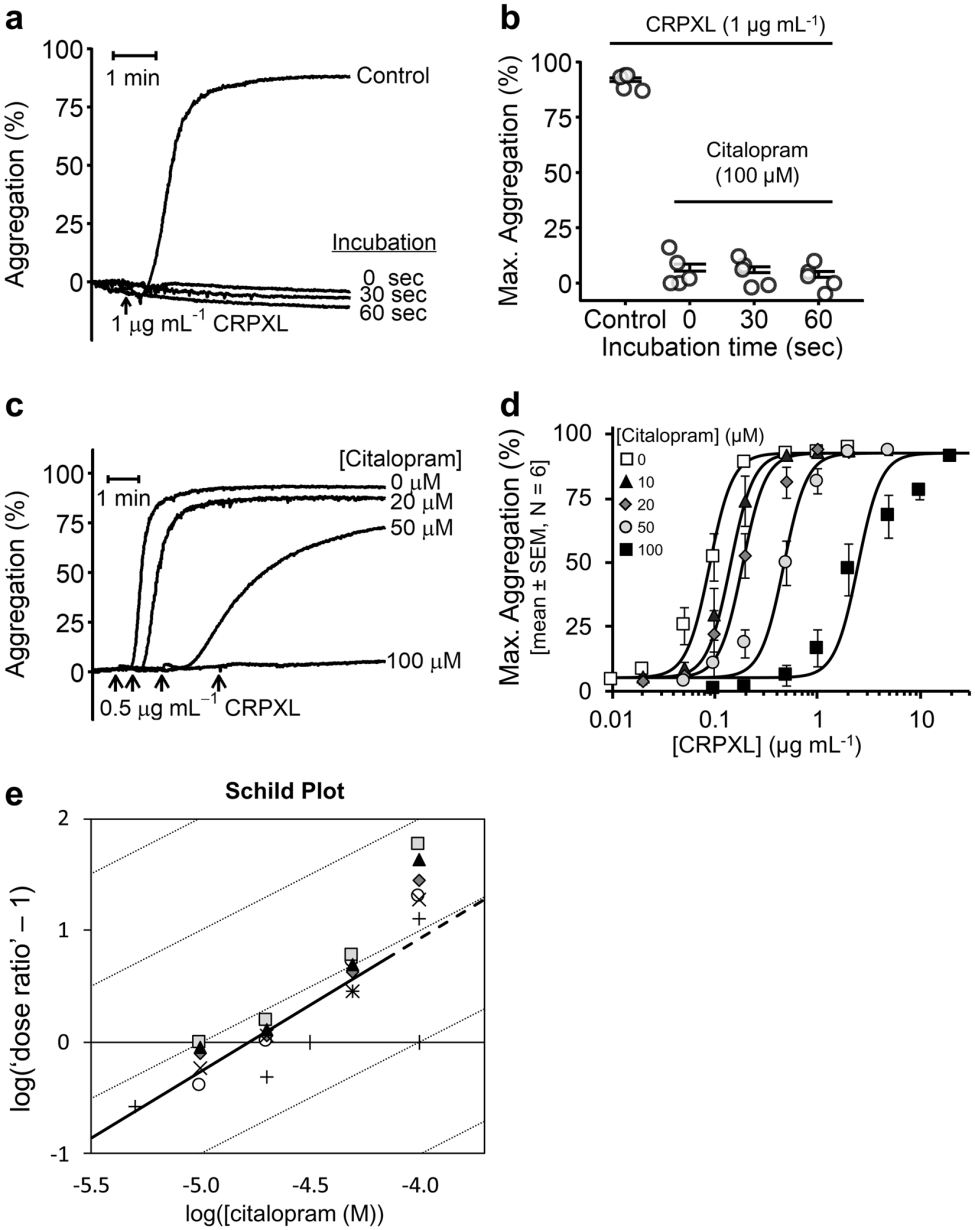
Citalopram instantly and competitively inhibits CRPXL-induced platelet aggregation. (**a**) Representative aggregation traces for either untreated platelets, or platelets pre-incubated with 100 µM citalopram for either 30 or 60 seconds before stimulation with cross-linked collagen-related peptide CRPXL (1 µg mL^−1^). 0 seconds represents simultaneous addition of citalopram (100 µM) and CRPXL. (**b**) Effect of varying pre-incubation times on the inhibitory effect of citalopram. Following CRPXL (1 µg mL^−1^) addition, the maximum extent of aggregation over 6 min (Max. Aggregation) was quantified (N = 4 blood donors). (**c**) Example aggregation traces of platelets pre-incubated with citalopram (0, 20, 50, & 100 µM) for approximately 5 min, before stimulation with CRPXL (0.5 µg mL^−1^). Arrowheads indicate time points of CRPXL addition. (**d**) Agonist concentration-response curves demonstrate how the maximum extent of platelet aggregation (Max. Aggregation) induced by a range of CRPXL concentrations was inhibited by citalopram. Concentrations of citalopram below 10 µM demonstrate similar responses to untreated platelets and were omitted from the figure for clarity. 5 µM citalopram was only tested in a single donor and was therefore omitted from the figure. (**e**) Schild analysis was carried out on a range of citalopram concentrations (5, 10, 20, 50 & 100 µM). Diagonal grey lines have a slope of 1, which corresponds to data expected from a competitive antagonist. The solid line represents the Schild regression line (slope = 1.19 ± 0.09) generated from the data excluding the 100 µM data points. (N = 6 blood donors. Different donors are indicated by different symbols).

CRPXL-induced (1 µg mL^−1^) platelet aggregation was completely inhibited by citalopram (100 µM) following either short pre-incubation times (30, 60 seconds) or on simultaneous addition with CRPXL (i.e., 0 seconds pre-incubation) (Fig. 6a,b). The same result was observed with collagen as an agonist (Supplementary Fig. S6). Following 5 min pre-incubations, citalopram inhibited CRPXL-induced platelet aggregation in a concentration-dependent manner (Fig. 6c,d). Pre-incubating platelets with 20 µM and 50 µM citalopram caused 2.1-fold and 5.3-fold rightward shifts in agonist-response curves, respectively (Fig. 6d). Schild analysis (Supplementary Analysis 3) for citalopram concentrations between 5 and 50 µM gave a Schild slope of 1.19 ± 0.09 (N = 6), a result consistent with competitive antagonism. The pA_2_ value was 4.79 ± 0.07 indicating a dissociation constant (K_d_) of approximately 16 µM. However, 100 µM citalopram caused a 25-fold rightward shift in the agonist-response curve (Fig. 6d). Inclusion of this concentration into the Schild analysis increased the Schild slope to 1.60 ± 0.05 (Fig. 6e). In three experiments, at 200 µM citalopram, there was no response to CRPXL (highest concentration tested = 20 µg mL^−1^).

These data suggest that at concentrations up to approximately 50 µM, citalopram inhibits CRPXL-induced platelet aggregation in a manner consistent with a competitive mechanism of action. Above this concentration, this pattern breaks down as may be predicted since, at these higher concentrations, citalopram will also exert inhibitory effects via its action on CalDAG-GEFI/Rap1.

## Discussion

We have previously shown that citalopram-induced inhibition of platelet function is not caused by blockade of SERT-dependent 5-HT uptake into platelets^10^. The aim of this study was to identify putative SERT-independent mechanisms of platelet inhibition by citalopram. Specifically, we have characterised the effects of citalopram on two distinct processes: (1) Rap1 activation and (2) GPVI receptor function.

In platelets and neutrophils, activation of both TP and PAF receptors respectively induces Ca^2+^ release from intracellular stores via G protein-mediated activation of phospholipase Cβ (PLCβ)^29–31^. In both cell types, citalopram failed to inhibit either U46619- or PAF-induced Ca^2+^ release indicating that the signalling pathways from receptor to elevated [Ca^2+^]_cyt_ were unaffected by the drug. By contrast, citalopram did block downstream Rap1 activation and cell function. Similarly, Tseng *et al*.^14^ reported that ADP-induced platelet aggregation was inhibited by citalopram, but not ADP-induced Ca^2+^ signalling. Notably, Ca^2+^-dependent Rap1 activation in both platelets and neutrophils is mediated by CalDAG-GEFI^15,21^. Our results from an *in vitro* fluorescence-based binding assay show citalopram inhibits CalDAG-GEFI-mediated nucleotide exchange of Rap1B. The recovery of CRPXL-, collagen- and U46619-induced aggregation after washing out citalopram (Fig. 5, Supplementary Figs S4 and S5) indicates that this inhibition by citalopram is reversible. We therefore propose that citalopram binds directly and reversibly to either CalDAG-GEFI, Rap1 or a complex of both, thereby inhibiting Rap1 activation.

Comparatively few studies have reported the *in vitro* effects of SSRIs on neutrophils. Although fluoxetine has previously been shown to inhibit some neutrophil functions^22^, we believe that ours is the first report of citalopram inhibiting human neutrophil function. Unlike platelets, neutrophils do not express SERT^32^. Therefore, our results provide further confirmation of a direct and SERT-independent mechanism of action of citalopram in neutrophils and, by extension, platelets.

We have previously reported that citalopram inhibited collagen-induced aggregation and phosphorylation of molecules in the GPVI signalling pathway^10^. We now report that citalopram also inhibits platelet aggregation induced by CRPXL, a GPVI-selective agonist, and reduces the binding of anti-GPVI antibodies to unstimulated platelets. One possible explanation for these results is a reduction in surface receptor number, either by shedding or internalisation. However, for a full agonist, a reduction in receptor number is predicted to reduce the observed potency of the agonist^33^, and this has previously been demonstrated for CRPXL-induced aggregation in platelets with 50% levels of GPVI^34^. Thus, the similarity in CRPXL responsiveness of untreated resuspended platelets (Fig. 5f, condition (3)) and citalopram-pre-treated resuspended platelets (Fig. 5f, condition (4)) suggests that little if any GPVI was lost from the platelet surface as a result of citalopram treatment. Moreover, our data show that inhibition of CRPXL-induced platelet aggregation by citalopram is both instantaneous in onset and fully reversible. Taken together, these data strongly support a reversible, competitive mechanism of action for citalopram, rather than a reduction in surface receptor expression. We therefore propose that citalopram binds directly to GPVI-FcRγ chain complex, thereby preventing collagen- and CRPXL-induced platelet activation.

Our proposal that citalopram exerts two distinct mechanisms of action is further supported by the observed inhibitory potencies of citalopram in our studies. The Schild analysis indicates that citalopram binds to GPVI/FcRγ chain with a K_d_ of approximately 16 µM. This is wholly consistent with data reported in our previous study^10^ showing that 20 µM citalopram caused an approximate 2-fold rightward shift of the collagen concentration-response curve but had no discernible effect on U46619-induced aggregation. *pIC*_50_ values for inhibition of GPVI-independent functions: aggregation induced by U46619 (4.15 ± 0.27) and ionomycin (3.98 ± 0.09); PAF-induced activation of neutrophil α_M_β_2_ (4.02 ± 0.15); adhesion of platelets (4.00 ± 0.07) and neutrophils (3.88 ± 0.04) to fibrinogen; and CalDAG-GEFI-dependent Rap1B activation (3.67 ± 0.32), are all consistent with citalopram binding to and inhibiting CalDAG-GEFI/Rap1B at a concentration of approximately 100 µM. This is further reflected by the Schild analysis showing a rightward shift in the CRPXL concentration-response curves consistent with competitive antagonism at citalopram concentrations up to 50 µM, whereas at higher concentrations the shift was greater (Fig. 6) caused by the combination of the two distinct inhibitory mechanisms outlined above.

Citalopram may inhibit platelets and neutrophils through other unidentified mechanisms that are distinct from SERT blockade. For example, Bonnin *et al*., (2012)^35^ have proposed that (*R*)-citalopram, the lower potency isomer^36^, may act via the orphan sigma-1 receptor. However, as we have previously noted, this is unlikely to be the mechanism responsible for the action of citalopram in platelets^10^.

**Figure 7:**
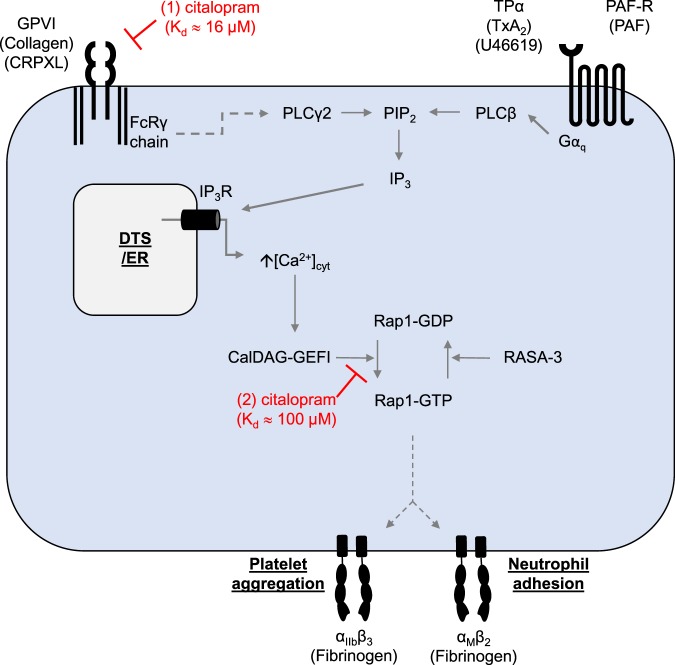
Two novel, putative mechanisms of action for citalopram-induced platelet and neutrophil inhibition. (1) In platelets, citalopram binds to the GPVI/FcRγ chain complex (K_d_ ≈ 16 µM), thereby acting as a competitive antagonist and blocking receptor activation by the GPVI agonists CRPXL and collagen. This prevents activation of phospholipase Cγ2 (PLCγ2), generation of inositol trisphosphate (IP_3_), activation of IP_3_ receptors (IP_3_R) and subsequent release of calcium from the dense tubular system (DTS) or endoplasmic reticulum (ER) into the cytoplasm. (2) In both platelets and neutrophils, at higher concentrations, citalopram interacts with CalDAG-GEFI/Rap1B (K_d_ ≈ 100 µM), thereby inhibiting the Ca^2+^- and CalDAG-GEFI-dependent conversion of Rap1B-GDP to Rap1B-GTP. (In platelets, Ras GTPase-activating protein 3 (RASA-3) mediates the conversion of Rap1B-GTP to Rap1B-GDP^51^). This model explains how citalopram can selectively disrupt GPVI-dependent platelet activation at concentrations between 20 and 50 µM, while inhibiting the CalDAG-GEFI/Rap1 signalling axis at concentrations above 50 µM in both platelets and neutrophils.

In summary, we propose a model (Fig. 7) in which citalopram binds to two distinct molecular targets: (1) GPVI/FcRγ chain (K_d_ ≈ 16 µM) and (2) CalDAG-GEFI/Rap1B (K_d_ ≈ 100 µM). This model predicts that citalopram would selectively disrupt GPVI-dependent platelet activation at concentrations between 20 and 50 µM, and above 50 µM, it would also inhibit Ca^2+^-dependent functions mediated through CalDAG-GEFI. These two novel, putative and distinct inhibitory mechanisms of action: (1) competitive antagonism of GPVI-FcRγ chain inhibition and (2), inhibition of CalDAG-GEFI-mediated nucleotide exchange of Rap1B, both need much higher concentrations of citalopram than are required to inhibit SERT, its primary mechanism of action. Hence, these effects are unlikely to be of clinical significance^10^, either as a cause of reported bleeding complications^37–41^, or as a strategy for reducing cardiovascular disease. Further studies will be required to confirm these putative mechanisms. However, if confirmed, citalopram may prove to be a useful investigative tool for the study of CalDAG-GEFI, Rap1, and GPVI signalling, as well as a practical chemical starting point for the discovery of more selective and potent inhibitors. A potent, selective GPVI antagonist could be a potentially useful anti-thrombotic agent and inhibitors of CalDAG-GEFI/Rap1 may have a wide range of uses in haematopoietic cells.

## Material and Methods

### Materials

Prostaglandin E_1_ (PGE_1_), indomethacin, U46619, citric acid, trisodium citrate, ionomycin, glycerol, dextran-500, Nonidet P-40 (NP-40), fibrinogen, p-nitrophenyl phosphate (pNPP) and Percoll^®^ were from Sigma (Poole, U.K.). Ethylene glycol-bis(2-aminoethyl ether)-*N*,*N*,*N’*,*N’*-tetraacetic acid (EGTA) was from Calbiochem (Nottingham, U.K.). BODIPY-FL-GDP, dithiothreitol, fluorescein (FITC)-conjugated anti-CD15 and allophycocyanin (APC)-conjugated anti-CD11b/CD18 antibodies were from Thermo Fisher Scientific, (Loughborough, U.K.). Alexa488-conjugated anti-mouse F(ab)_2_ was from Jackson ImmunoResearch (Ely, U.K.). Murine IgG_2a_κ isotype control antibody was from BioLegend (London, U.K.). (*RS*)-citalopram and PAF were from Cambridge Bioscience (Cambridge, U.K.). Horm^®^ collagen was from Takeda (Linz, Austria). Bovine serum albumin (BSA) was from GE Healthcare (Buckinghamshire, U.K.). Fura-2 (AM) was from TEFLabs (Cambridge, U.K.). CRPXL was synthesised in the laboratory of Professor Richard Farndale (University of Cambridge, U.K.).

### Blood donation

Donation of fresh blood from healthy, consenting human volunteers was approved by the University of Cambridge Human Biology Research Ethics Committee (Ref: HBREC.2015.18). Prior to any blood donation, informed consent was obtained from each blood donor. The consent form was signed by both the donor and one of the project supervisors (G.E.J. or S.O.S.). A fresh consent form is signed annually. On the occasion of each donation of blood, a donation record form was signed by both the donor and the phlebotomist (G.E.J., S.O.S. or H.G.R.). Collection of blood from donors, its use and subsequent disposal were performed in accordance with relevant guidelines and regulations. Blood was drawn into 50 mL syringes containing trisodium citrate (final concentration of 11 mM in blood).

### Washed platelet preparation

Citrated blood was centrifuged (500 × g, 5 min) to obtain platelet-rich plasma (PRP). Following addition of PGE_1_ (final concentration of 1 μM), PRP was centrifuged (900 × g, 15 min) and the resulting platelet pellet resuspended in a modified calcium-free Tyrode’s buffer (CFT; 137 mM NaCl, 11.9 mM NaHCO_3_, 0.4 mM NaH_2_PO_4_, 2.7 mM KCl, 1.1 mM MgCl_2_, 5.6 mM glucose; pH = 7.4). Platelet counts were adjusted to 2 × 10^8^ mL^−1^ using a Z2 Coulter particle counter (Beckman Coulter, High Wycombe, U.K.).

### Neutrophil preparation

Citrated blood was mixed 2:1 with a saline solution of dextran-500 (final concentration of 1% [w/v]) and left for 30 min to allow red blood cell (RBC) sedimentation, whilst retaining white blood cells (WBCs) within the PRP. WBC-rich PRP was aspirated and layered over a discontinuous density gradient of Percoll^®^ (1.5 mL of 1.088 g mL^−1^ Percoll^®^, carefully layered on top of 1.5 mL of 1.100 g mL^−1^ Percoll^®^). Samples were centrifuged (600 × g, 20 min) to separate granulocytes from the lower-density platelets, lymphocytes and monocytes. The isolated granulocyte band was aspirated, washed with phosphate-buffered saline (PBS), centrifuged (300 × g, 5 min) and resuspended in CFT. The cell concentration was adjusted to 1 × 10^6^ mL^−1^ using a Z2 Coulter particle counter (Beckman Coulter, High Wycombe, U.K.).

### Platelet aggregometry

Platelet aggregation was measured by turbidimetric aggregometry as previously described^10,42^ using two Aggregation Remote Analyzer Modules (AggRAM) with HemoRAM software (v1.2) (Helena Biosciences, Newcastle, U.K.). Washed platelets (WP) (247.5 µL, 2 × 10^8^ mL^−1^) were aliquoted into glass cuvettes containing magnetic stir bars. 2.5 µL agonist was added and aggregation recorded at 37 °C with a stir speed of 1,000 rpm. The maximum extent of aggregation over 6 min was determined as previously described^43^.

### Monitoring cytosolic calcium concentration

Changes in the cytosolic concentration of Ca^2+^ ([Ca^2+^]_cyt_) were monitored using the fluorescent Ca^2+^ indicator, Fura-2. PRP or isolated neutrophils were incubated for 30 min at 37 °C with 2.5 µM Fura-2 AM. With PRP, WP preparation was subsequently continued as described above. Fura-2-loaded neutrophils were centrifuged (300 × g, 5 min) and resuspended in fresh CFT to 1 × 10^6^ mL^−1^. Fura-2 fluorescence was measured using a Cairn Optoscan Spectrophotometer and Acquisition Engine (v1.1.7) (Cairn Research, Faversham, U.K.) in stirred 1.2 mL samples of WP or neutrophils at 37 °C. Following chelation of extracellular Ca^2+^ by EGTA (10 mM), agonist-induced changes in [Ca^2+^]_cyt_ were monitored for 3 min. [Ca^2+^]_cyt_ was calculated using the method of Grynkiewicz *et al*.^44^. The agonist-induced response (peak [Ca^2+^]) was obtained by subtracting the basal [Ca^2+^]_cyt_ from the peak signal.

### Rap1 activation and Western Blot

Rap1 activation was measured using an Active Rap1 Pull-Down and Detection Kit (Thermo Fisher Scientific, Loughborough, U.K.). 247.5 µL of either WP (2 × 10^8^ mL^−1^) or neutrophils (1 × 10^6^ mL^−1^) were stimulated in AggRAM aggregometers for 1 min with either CRPXL (0.5 µg mL^−1^), U46619 (0.2 µM) or PAF (1 µM). Reactions were terminated with 1:1 lysis/binding/wash buffer (LBW: 25 mM Tris HCl, 150 mM NaCl, 5 mM MgCl_2_, 1% [v/v] NP-40, 5% [v/v] glycerol, pH = 7.2). Lysates were put on ice for 5 min, followed by centrifugation (8,000 × g, 1 min) to remove cellular debris. For total Rap1 quantification, 20 µL of each sample lysate was aliquoted into 1:1 Laemmli buffer (final concentrations: 62.5 mM Tris HCl, 1% [v/v] glycerol, 2% [w/v] SDS, 2.5% [v/v] mercaptoethanol, 0.025% [w/v] brilliant blue). For active Rap1 (i.e., Rap1-GTP) quantification, the remaining 480 µL sample was aliquoted into filter spin cups containing 100 µL of 50% glutathione-agarose beads and 20 µg of GST-RalGDS-RBD fusion protein. Samples were briefly vortexed then incubated with gentle rocking (1 hour, 4 °C). Samples were centrifuged (8,000 × g, 1 min) and washed 3 times with 400 µL LBW, before the addition of 50 µL Laemmli buffer. Samples were then centrifuged (8,000 × g, 2 min) through the spin cups to obtain bead-free samples for Western blot analysis.

Total Rap1 and Rap1-GTP samples underwent SDS PAGE and Western blot analysis. Samples were added to 10 well 4–12% pre-cast NuPage Bis-Tris gels (Invitrogen, Paisley, U.K.), followed by SDS/PAGE separation and transfer to PVDF membranes (Millipore, Watford, U.K.). Membranes were incubated with Rap1 primary antibodies (Thermo Fisher Scientific, Loughborough, U.K) followed by incubation with HRP-conjugated secondary antibodies (Dako, Ely, U.K.). Enhanced chemiluminescence (ECL) and X-ray hyperfilm^®^ (Amersham Biosciences, Buckinghamshire, U.K.) were used to detect protein bands. Developed X-ray films were scanned, and unprocessed images analysed using ImageJ (v1.50) as follows: identical areas (height (100) × width (150) = 15,000 pixels) were drawn around each protein band and the density of all pixels (scale 0–255 each) summed. The density of each area was therefore quantified on a scale from 0 (totally white) to 3,825,000 (totally black). Values are presented as % black. Uncropped images of X-ray films used for quantification of Rap1-GTP are shown in Supplementary Figs S7 and S8.

### Monitoring Rap1 nucleotide exchange activity

The rate of Rap1 nucleotide exchange was measured using a fluorescence-based *in vitro* enzyme assay^17,45,46^. 100 µL of reaction buffer (20 mM Tris base, 150 mM NaCl, 5 mM MgCl_2_, 2 mM dithiothreitol, 10% [v/v] glycerol, 0.08% [v/v] NP-40, 1 µM Rap1B, 0.1 µM BODIPY-FL-GDP, pH = 7.5) was aliquoted into wells of a Nunc F96 well, black, flat-bottomed plate and the baseline fluorescence intensity (F.I.) recorded (excitation 485 nm; emission 520 nm) for 3 min with a Fluostar Optima plate reader (BMG Labtech, Aylesbury, U.K.). Measurements were halted and CalDAG-GEFI (0.3 µM) added to increase the rate of BODIPY-FL-GDP exchange onto Rap1, thereby increasing F.I. Recording was resumed and the average F.I. prior to CalDAG-GEFI addition subtracted from the final F.I. 20 min after the addition of CalDAG-GEFI (ΔF.I.).

Rap1B and CalDAG-GEFI were cloned from human genes into a protein expression vector p15LIC2 6xHis, which was purified in *E*. *coli*, as previously described^17^. Both proteins contained a C-terminal truncation (p.(Lys168_Leu184del) and p.(Ala552_Leu609del), respectively) which removed disordered regions to improve stability during the purification process, while leaving all the functional domains intact. Protein sequences (native and recombinant mutants) for both Rap1B and CalDAG-GEFI are shown in Supplementary Fig. S9.

### Neutrophil integrin α_M_β_2_ activation

Neutrophil integrin α_M_β_2_ (Mac-1, CD11b/CD18) activation was measured using an APC-conjugated monoclonal antibody, which binds the activated epitope of integrin α_M_ (CD11b). Neutrophils (100 µL, 1 × 10^6^ mL^−1^) were incubated with the active CD11b antibody in the absence of light (5 min, 4 °C). Samples were fixed with 2% [v/v] paraformaldehyde and neutrophils gated by forward scatter (FSC), side scatter (SSC) and CD15^+^ criteria. The median fluorescence intensity (M.F.I.) of activated CD11b from 30,000 gated events was then determined for each sample using an Accuri^TM^ C6 flow cytometer (BD Bioscience, Oxford, U.K.).

### Neutrophil adhesion

The adhesion of neutrophils to fibrinogen under static conditions was quantified by adapting a protocol developed to measure levels of cell-derived acid phosphatase (EC 3.1.3.2)^47^. Immulon-2HB 96 flat-bottom well plates (Thermo Fisher Scientific, Loughborough, U.K.) were incubated overnight at 4 °C with 100 μL of fibrinogen (10 μg mL^−1^ in saline). Excess ligand was discarded, and wells blocked with 175 μL BSA (5% [w/v] in CFT) for 1 hour. Wells were washed three times with BSA (0.1% [w/v] in CFT). 50 μL of isolated neutrophils (4.00 × 10^6^ mL^−1^) were added to wells and left for 1 hour at room temperature. Samples were discarded, and the wells washed as before, followed by the addition of 150 μL of citrate lysis buffer (3.53 mM pNPP, 71.4 mM trisodium citrate, 28.55 mM citric acid, 0.1% [v/v] Triton X-100; pH 5.4). After 1 hour, 100 μL of 2 M NaOH was added and absorbance measured at 405 nm with a Sunrise^TM^ plate reader (Tecan, Reading, U.K.).

### Lactate dehydrogenase cytotoxicity assay

LDH release from neutrophils was measured to determine drug-induced cytotoxicity and cytolysis, using a Pierce LDH Activity Assay Kit (Thermo Fisher Scientific, Loughborough, U.K.). Neutrophils (250 µL, 1 × 10^6^ mL^−1^) were centrifuged (8,000 × g, 1 min) and 50 µL of supernatant was aliquoted into wells of an Immulon-2HB 96-well flat-bottom plate. 50 µL of the proprietary reaction mixture (Thermo Fisher, product code: 1862887) was added to each well for 30 min. 50 µL of the proprietary stop solution (Thermo Fisher, product code: 1862880) terminated the reaction and background absorbance at 680 nm was subtracted from the absorbance at 490 nm. Measurements were made using a Sunrise^TM^ plate reader (Tecan, Reading, U.K.).

### Antibody Binding to Glycoprotein VI

The binding of antibodies to dimeric or total (dimeric and monomeric) platelet GPVI was quantified as previously described using 204-11 Fab^48^ and HY-101^49^ antibodies, respectively. WP (2.50 × 10^7^ mL^−1^) were incubated for 10 min with either HY-101 (5 µg mL^−1^) or 204-11 Fab (10 µg mL^−1^). Murine IgG_2a_κ or Fab were used as corresponding isotype controls, respectively. 5 µg mL^−1^ of Alexa488-conjugated anti-mouse F(ab)_2_ was subsequently added and incubated in the dark for 10 min. Samples were diluted 1:8 in CFT and the M.F.I. measured using an Accuri^TM^ C6 flow cytometer (BD Bioscience, Oxford, U.K.).

### Data and statistical analysis

Concentration-response data were modelled using a four-parameter logistic (4PL) model^43,50^:

$${R}_{PRED}=\frac{Min-Max}{1+{([A]/{10}^{-p{A}_{50}})}^{{n}_{H}}}+Max$$

Where: *R*_*PRED*_ = predicted response (dependent variable); [*A*] = agent concentration (independent variable); *Min* = response when [*A*] = 0; *Max* = response when [*A*] = ∞; *pA*_50_ = -log [*A*] (expressed in units of mol L^−1^, or g mL^−1^ for CRPXL) when *R*_*PRED*_ = (*Max* + *Min*)/2; *n*_*H*_ = Hill coefficient. When *A* is an inhibitor, the *pA*_50_ is the *pIC*_50_. Unless otherwise stated, fitting was performed using minimisation of least squares with the Solver function in Microsoft^®^ Excel. Data are presented as mean ± standard error of the mean (SEM). Figures were generated using either R 3.3.2 (The R Foundation for Statistical Computing, Vienna, Austria) or Microsoft^®^ Excel. Additional statistical analyses are presented in Supplementary Methods.

## Electronic supplementary material

Supplementary Information

## References

[1] Bullard, I. *Prescriptions Dispensed in the Community*, *England 2006 to 2016*. (NHS Digital, 2017).

[2] Blakely RD (1991). Cloning and expression of a functional serotonin transporter from rat brain. Nature.

[3] Hoffman BJ, Mezey E, Brownstein MJ (1991). Cloning of a serotonin transporter affected by antidepressants. Science.

[4] Hyttel J (1982). Citalopram pharmacological profile of a specific serotonin uptake inhibitor with antidepressant activity. Prog Neuropsychopharmacol Biol Psychiatry.

[5] Owens MJ (2004). Selectivity of antidepressants: From the monoamine hypothesis of depression to the SSRI revolution and beyond. J Clin Psychiatry.

[6] Goubau C, Buyse GM, Di Michele M, Van Geet C, Freson K (2013). Regulated granule trafficking in platelets and neurons: A common molecular machinery. Eur J Paediatr Neurol.

[7] Maurer-Spurej E, Pittendreigh C, Solomons K (2004). The influence of selective serotonin reuptake inhibitors on serotonin metabolism in human platelets. Thromb Haemost.

[8] Lopez-vilchez I (2017). Escitalopram Impairs Thrombin-Induced Platelet Response, Cytoskeletal Assembly and Activation of Associated Signalling Pathways. Thromb Haemost.

[9] Tseng YL (2010). A selective serotonin reuptake inhibitor, citalopram, inhibits collagen-induced platelet aggregation and activation. Thromb Res.

[10] Roweth HG (2018). Citalopram inhibits platelet function independently of SERT-mediated 5-HT transport. Sci Rep.

[11] Carneiro AMD, Cook EH, Murphy DL, Blakely RD (2008). Interactions between integrin αIIbβ3 and the serotonin transporter regulate serotonin transport and platelet aggregation in mice and humans. J Clin Invest.

[12] Galan AM (2009). Serotonergic mechanisms enhance platelet-mediated thrombogenicity. Thromb Haemost.

[13] Jung SM (2012). Constitutive dimerization of glycoprotein VI (GPVI) in resting platelets is essential for binding to collagen and activation in flowing blood. J Biol Chem.

[14] Tseng YL, Chiang ML, Lane HY, Su KP, Lai YC (2013). Selective serotonin reuptake inhibitors reduce P2Y12 receptor-mediated amplification of platelet aggregation. Thromb Res.

[15] Crittenden JR (2004). CalDAG-GEFI integrates signaling for platelet aggregation and thrombus formation. Nat Med.

[16] Stefanini L, Bergmeier W (2016). RAP1-GTPase signaling and platelet function. J Mol Med.

[17] Cook AA (2018). Calcium-induced structural rearrangements release autoinhibition in the Rap-GEF, CalDAG-GEFI. J Cell Biol.

[18] Lee HS, Lim CJ, Puzon-McLaughlin W, Shattil SJ, Ginsberg MH (2009). RIAM activates integrins by linking talin to Ras GTPase membrane-targeting sequences. J Biol Chem.

[19] Moser M, Legate KR, Zent R, Fassler R (2009). The tail of integrins, talin, and kindlins. Science.

[20] M’Rabet, L. *et al*. Activation of the small GTPase Rap1 in human neutrophils. *Blood* **92**, 2133–2140 (1998). 9731072

[21] Bergmeier W (2007). Mice lacking the signaling molecule CalDAG-GEFI represent a model for leukocyte adhesion deficiency type III. J Clin Invest.

[22] Strümper D (2003). Effects of antidepressants on function and viability of human neutrophils. Anesthesiology.

[23] Burkhart JM (2012). The first comprehensive and quantitative analysis of human platelet protein composition allows the comparative analysis of structural and functional pathways. Blood.

[24] Filippi MD (2015). Leukocyte transcellular diapedesis: Rap1b is in control. Tissue Barriers.

[25] Kumar S (2014). The small GTPase Rap1b negatively regulates neutrophil chemotaxis and transcellular diapedesis by inhibiting Akt activation. J Exp Med.

[26] Caron E, Self AJ, Hall A (2000). The GTPase Rap1 controls functional activation of macrophage integrin αMβ2 by LPS and other inflammatory mediators. Curr Biol.

[27] Yakubenko VP (2001). Identification of the Binding Site for Fibrinogen Recognition Peptide γ383-395 within the αMI-Domain of Integrin αMβ2. J Biol Chem.

[28] Poulter NS, Pollitt AY, Owen DM, Gardiner EE, Andrews RK (2017). Clustering of glycoprotein VI (GPVI) dimers upon adhesion to collagen as a mechanism to regulate GPVI signaling in platelets. J Thromb Haemost.

[29] Kinsella BT, O’Mahony DJ, Fitzgerald GA (1997). The human thromboxane A2 receptor alpha isoform (TP alpha) functionally couples to the G proteins Gq and G11 *in vivo* and is activated by the isoprostane 8-epi prostaglandin F2 alpha. J Pharmacol Exp Ther..

[30] Paul BZS, Jin J, Kunapuli SP (1999). Molecular Mechanism of Thromboxane A 2 induced Platelet Aggregation. J Biol Chem.

[31] Honda Z, Ishii S, Shimizu T (2002). Platelet-Activating Factor Receptor. J Biochem.

[32] Herr N, Bode C, Duerschmied D (2017). The Effects of Serotonin in Immune Cells. Front Cardiovasc Med.

[33] Black JW, Leff P, Shankley NP, Wood J (1985). An operational model of pharmacological agonism: the effect of E/[A] curve shape on agonist dissociation constant estimation. Br J Pharmacol.

[34] Snell DC (2002). Differential effects of reduced glycoprotein VI levels on activation of murine platelets by glycoprotein VI ligands. Biochem J.

[35] Bonnin A, Zhang L, Blakely RD, Levitt P (2012). The SSRI citalopram affects fetal thalamic axon responsiveness to netrin-1 *in vitro* independently of SERT antagonism. Neuropsychopharmacology.

[36] Owens MJ, Knight DL, Nemeroff CB (2001). Second-generation SSRIs: human monoamine transporter binding profile of escitalopram and R-fluoxetine. Biol Psychiatry.

[37] van Walraven C, Mamdani MM, Wells PS, Williams JI (2001). Inhibition of serotonin reuptake by antidepressants and retrospective cohort study. Br Med J.

[38] Dalton SO (2003). Use of selective serotonin reuptake inhibitors and risk of upper gastrointestinal tract bleeding. Arch Intern Med.

[39] Opatrny L, Delaney JAC, Suissa S (2008). Gastro-intestinal haemorrhage risks of selective serotonin receptor antagonist therapy: A new look. Br J Clin Pharmacol.

[40] Dall M (2009). An association between selective serotonin reuptake inhibitor use and serious upper gastrointestinal bleeding. Clin Gastroenterol Hepatol.

[41] Hackam DG, Mrkobrada M (2012). Selective serotonin reuptake inhibitors and brain hemorrhage. Neurology.

[42] Jarvis GE (2004). Platelet aggregation: turbidimetric measurements. Methods Mol Biol.

[43] Jarvis GE, Humphries RG, Robertson MJ, Leff P (2000). ADP can induce aggregation of human platelets via both P2Y1 and P2T receptors. Br J Pharmacol.

[44] Grynkiewicz G, Poenie M, Tsien RY (1985). A new generation of Ca2+ indicators with greatly improved fluorescence properties. J Biol Chem.

[45] Lozano ML (2016). Novel mutations in RASGRP2, which encodes CalDAG-GEFI, abrogate Rap1 activation, causing platelet dysfunction. Blood.

[46] Ren J, Cook AA, Bergmeier W, Sondek J (2016). A negative-feedback loop regulating ERK1/2 activation and mediated by RasGPR2 phosphorylation. Biochem Biophys Res Commun.

[47] Bellavite P (1994). A colorimetric method for the measurement of platelet adhesion in microtiter plates. Anal Chem.

[48] Jung SM, Tsuji K, Moroi M (2009). Glycoprotein (GP) VI dimer as a major collagen-binding site of native platelets: direct evidence obtained with dimeric GPVI-specific Fabs. J Thromb Haemost.

[49] Chen H, Locke D, Liu Y, Liu C, Kahn ML (2002). The platelet receptor GPVI mediates both adhesion and signaling responses to collagen in a receptor density-dependent fashion. J Biol Chem.

[50] DeLean A, Munson PJ, Rodbard D (1978). Simultaneous analysis of families of sigmoidal curves: application to bioassay, radioligand assay, and physiological dose-response curves. Am J Physiol.

[51] Stefanini L (2015). RASA3 is a critical inhibitor of RAP1-dependent platelet activation. J Clin Invest.

