# Supplementary material for: Two novel, putative mechanisms of action for citalopram-induced platelet inhibition
Source: Sci Rep. 2018 Nov 12;8:16677. doi: 10.1038/s41598-018-34389-5 (PMC6232110; doi:10.1038/s41598-018-34389-5)
Supplement: Supplementary file 1 — Supplementary Information [file 41598_2018_34389_MOESM1_ESM.docx]

**Two novel, putative mechanisms of action for citalopram-induced platelet inhibition**

Harvey G. Roweth^1^, Aaron A. Cook^2^, Masaaki Moroi^3^, Arkadiusz M. Bonna^3^, Stephanie M. Jung^3^, Wolfgang Bergmeier^2^, Stewart O. Sage^1^ and Gavin E. Jarvis^1^.

**Supplementary Information**

1. Supplementary Analyses SA1 to SA3
   1. SA1: Rap1 activation in platelets (pp. 2-3)
   2. SA2: Rap1 activation in neutrophils (pp. 4-5)
   3. SA3: Schild Analysis (p. 6)
2. Supplementary Figures S1 to S9
   1. S1: CRPXL-induced and U46619-induced calcium store release (p. 7)
   2. S2: Citalopram inhibits ionomycin-induced platelet aggregation (p. 8)
   3. S3: Citalopram is not cytotoxic to neutrophils (p. 9)
   4. S4: Citalopram inhibition of collagen-induced platelet activation is reversible (p. 10)
   5. S5: Citalopram inhibition of U46619-induced platelet activation is reversible (p. 11)
   6. S6: Instantaneous platelet inhibition by citalopram (p. 12)
   7. S7: Uncropped images of X-ray films of Western blots showing levels of Rap1-GTP and total Rap1 in platelet lysates (pp. 13-14)
   8. S8: Uncropped images of X-ray films of Western blots showing levels of Rap1-GTP and Rap1 in neutrophil lysates (pp. 15-16)
   9. S9: Protein sequences for recombinant CalDAG-GEFI and Rap1B (p. 17)

**Supplementary Analysis 1:**

**Citalopram inhibits Rap1 activation in platelets**

Rap1 signalling data from platelets were analysed using a bespoke multiple linear regression model. The principal hypothesis to be tested was whether citalopram altered agonist-induced levels of Rap1-GTP.

Levels of Rap1-GTP were estimated following Western Blot by quantifying grey levels in a uniform rectangular area using densitometry as described in the main Methods section. Individual values were transformed to represent the extent of grey on a scale from 0% (total white) to 100% (total black).

The experiment was repeated 4 times, using a different donor on each occasion. For each experiment, the following levels of Rap1-GTP were measured:

1. Background (i.e., no platelets)
2. Basal (untreated platelets)
3. CRPXL (0.5 µg mL^-1^)-treated platelets
4. CRPXL (0.5 µg mL^-1^)-treated platelets in the presence of citalopram (200 µM)
5. U46619 (0.2 µM)-treated platelets
6. U46619 (0.2 µM)-treated platelets in the presence of citalopram (200 µM)

Data were modelled to accommodate a varying background signal from the four different experiments, basal levels of Rap1-GTP in platelets, the effect of CRP and U46619 and the effect of citalopram on levels of agonist-induced Rap1-GTP.

Data were fitted to the following model:

*PRED_i_* = β_0_ + β_1_*x*_1_ + β_2_*x*_2_ + β_3_*x*_3_ + β_4_*x*_2_*x*_4_ + β_5_*x*_3_*x*_5_ + β_6_*x*_6_ + β_7_*x*_7_ + β_8_*x*_8_ + (- β_6_ - β_7_ - β_8_)*x*_9_ + ε*_i_*

Parameters were as follows:

1. β_0_ = Background grey (averaged across N = 4 experiments)
2. β_1_ = Additional grey from basal platelets
3. β_2_ = CRPXL-induced effect above basal
4. β_3_ = U46619-induced above basal
5. β_4_ = Citalopram effect in CRP-treated platelets
6. β_5_ = Citalopram effect in U46619-treated platelets
7. β_6_ = Difference between mean background and experiment 1
8. β_7_ = Difference between mean background and experiment 2
9. β_8_ = Difference between mean background and experiment 3
10. (- β_6_ - β_7_ - β_8_) = Difference between mean background and experiment 4

Variables were as follows:

1. *DV_i_* = dependent variable, i.e., the observed level of grey for data point *i*
2. *PRED_i_* = the predicted level of grey for data point *i*
3. *x*_1_ = 0 for background (no platelets), otherwise 1 for all platelet samples
4. *x*_2_ = 1 when CRPXL present, otherwise 0
5. *x*_3_ = 1 when U46619 present, otherwise 0
6. *x*_4_ = 1 when CRPXL and citalopram present, otherwise 0
7. *x*_5_ = 1 when U46619 and citalopram present, otherwise 0
8. *x*_6_ = 1 for experiment 1, otherwise 0
9. *x*_7_ = 1 for experiment 2, otherwise 0
10. *x*_8_ = 1 for experiment 3, otherwise 0
11. *x*_9_ = 1 for experiment 4, otherwise 0

Error residual for data point *i*: ε*_i_* = *PRED_i_* – *DV_i_*

The sum of the squared error residuals was minimised using the Solver function in Microsoft® Excel.

The principal (*a priori*) hypothesis (H_0_: citalopram effect = 0; H_1_: citalopram effect ≠ 0) was evaluated using an F-test by comparing the full model (9 parameters) with a partial model (7 parameters) where β_4_ = β_5_ = 0. Results from this analysis strongly indicate that citalopram inhibited CRPXL- and U46619-induced levels of Rap1-GTP (*P* = 7.5 × 10^-9^; F = 83.4; ν_1_ = 2, ν_2_ = 15).

A secondary post-hoc hypothesis was tested to evaluate whether there were any basal levels of Rap1-GTP in platelets and whether citalopram reduced levels of Rap1-GTP to basal levels. An F-test was used to compare the full model to a partial model in which β_1_ = 0, β_4_ = -β_2_ and β_5_ = -β_3_. The result (*P* = 0.42; F = 1.00; ν_1_=3, ν_2_=15) provided no evidence of basal levels of Rap1-GTP, and was consistent with citalopram abolishing the response induced by CRPXL and U46619.

These findings show that following U46619 stimulation, Rap1B activation is abolished by a concentration of citalopram (200 µM) that had no effect on Ca^2+^ release from intracellular stores.

**Supplementary Analysis 2:**

**Citalopram inhibits Rap1 activation in neutrophils**

Rap1 signalling data from neutrophils were analysed using a bespoke multiple linear regression model. The principal hypothesis to be tested was whether citalopram altered agonist-induced levels of Rap1-GTP.

Levels of Rap1-GTP were estimated following Western Blot by quantifying grey levels in a uniform rectangular area using densitometry as described in the main Methods section. Individual values were transformed to represent the extent of grey on a scale from 0% (total white) to 100% (total black).

The experiment was repeated 4 times, using a different donor on each occasion. For each experiment, the following levels of Rap1-GTP were measured:

1. Background (i.e., no neutrophils)
2. Basal (untreated neutrophils)
3. PAF (1 µM)-treated neutrophils
4. Citalopram (200 µM)-treated neutrophils
5. PAF (1 µM)-treated neutrophils in the presence of citalopram (200 µM)

Data were modelled to accommodate a varying background signal from the four different experiments, basal levels of Rap1-GTP in platelets, the effect of CRP and U46619 and the effect of citalopram on levels of agonist-induced Rap1-GTP.

Data were fitted to the following model:

*PRED_i_* = β_0_ + β_1_*x*_1_ + β_2_*x*_2_ + β_3_*x*_3_ + β_4_*x*_2_*x*_3_ + β_5_*x*_4_ + β_6_*x*_5_ + β_7_*x*_6_ + (- β_5_ - β_6_ - β_7_)*x*_7_ + ε*_i_*

Parameters were as follows:

1. β_0_ = Background grey (averaged across N = 4 experiments)
2. β_1_ = Additional grey from basal neutrophils
3. β_2_ = PAF-induced effect above basal
4. β_3_ = Citalopram-induced effect above basal
5. β_4_ = Citalopram effect in PAF-treated neutrophils
6. β_5_ = Difference between mean background and experiment 1
7. β_6_ = Difference between mean background and experiment 2
8. β_7_ = Difference between mean background and experiment 3
9. (- β_5_ - β_6_ - β_7_) = Difference between mean background and experiment 4

Variables were as follows:

1. *DV_i_* = dependent variable, i.e., the observed level of grey for data point *i*
2. *PRED_i_* = the predicted level of grey for data point *i*
3. *x*_1_ = 0 for background (no neutrophils), otherwise 1 for all neutrophil samples
4. *x*_2_ = 1 when PAF present, otherwise 0
5. *x*_3_ = 1 when citalopram present, otherwise 0
6. *x*_4_ = 1 for experiment 1, otherwise 0
7. *x*_5_ = 1 for experiment 2, otherwise 0
8. *x*_6_ = 1 for experiment 3, otherwise 0
9. *x*_7_ = 1 for experiment 4, otherwise 0

Error residual for data point *i*: ε*_i_* = *PRED_i_* – *DV_i_*

The sum of the squared error residuals was minimised using the Solver function in Microsoft® Excel.

The principal (*a priori*) hypothesis (H_0_: citalopram effect = 0; H_1_: citalopram effect ≠ 0) was evaluated using an F-test by comparing the full model (8 parameters) with a partial model (6 parameters) where β_3_ = β_4_ = 0. Results from this analysis strongly indicate that citalopram reduced PAF-induced levels of Rap1-GTP (*P* = 5.9 × 10^-6^; F = 38.7; ν_1_ = 2, ν_2_ = 12).

A secondary post-hoc hypothesis was tested to evaluate whether citalopram reduced levels of Rap1-GTP to basal levels. An F-test was used to compare the full model (8 parameters) to a partial model (6 parameters) in which β_3_ = 0 and β_4_ = -β_2_. The result (*P* = 0.0042; F = 8.92; ν_1_=2, ν_2_=12) suggests that citalopram did not completely abolish the effect of PAF.

These findings show that following PAF-induced Rap1 activation is substantially attenuated by a concentration of citalopram (200 µM) that had no effect on Ca^2+^ release from intracellular stores.

**Supplementary Analysis 3:**

**Schild Analysis of CRPXL-induced platelet aggregation inhibited by citalopram**

CRPXL-induced platelet aggregation was measured in the absence of citalopram and at citalopram concentrations ranging from 1 to 200 µM. In three experiments, 200 µM citalopram caused complete blockade of the response at all concentrations of CRPXL used. Since no agonist concentration-response data were obtained at 200 µM citalopram these data were not used in the subsequent Schild analysis.

Data for Schild analysis were obtained from six separate donors. For each donor, and for each concentration of citalopram, agonist concentration-response curve data were fitted using least squares minimisation to the four parameter logistic (4PL) equation (see Main Methods) to generate a Full Model. Data were then re-fitted with *Max*, *Min* and *n_H_* parameters constrained to be equal for all concentrations of citalopram from 0 to 100 µM (Partial Model) as would be expected with a competitive rightward shift. In only 1/6 experiments was there statistical evidence (using and F-test) of a difference in these parameters between different concentrations of citalopram (see Table).

| **Donor** | **Expt** | **N**  **collected** | **N**  **excluded** | **N**  **analysed** | ***Max***  **(constrained)** | ***Min***  **(constrained)** | ***n_H_***  **(constrained)** | ***pA*_50_**  **[citalopram] = 0** | ***P***  **(Full v Partial Models)** |
| --- | --- | --- | --- | --- | --- | --- | --- | --- | --- |
| #001 | 1 | 79 | 8  ([cit] = 200 µM)  1* | 70 | 90.6 | 5.5 | 4.9 | 7.08 | 0.53 |
| #009 | 2 | 87 | 0 | 87 | 94.2 | 3.6 | 3.4 | 6.93 | 1.00 |
| #052 | 3 | 80 | 0 | 80 | 90.6 | 4.1 | 2.5 | 7.36 | 0.001 |
| #068 | 4 | 72 | 0 | 72 | 92.8 | 3.6 | 2.5 | 6.99 | 0.23 |
| #072 | 5 | 89 | 3* | 86 | 93.9 | 7.4 | 5.7 | 6.90 | 0.99 |
| #076 | 6 | 84 | 0 | 84 | 93.4 | 6.9 | 4.2 | 7.04 | 0.53 |
| * these data points deviated substantially and unexpectedly from the model and were therefore excluded from the analysis as outliers | | | | | | | | | |

*pA*_50_ values from the constrained models were used to construct Schild plots (log (‘dose ratio’ - 1) *vs* log [citalopram]) for each experiment (see Fig. 6 in main paper). (Citalopram concentrations of 1 µM were omitted from this analysis as they had little to no effect on the agonist *pA*_50_.) This generated Schild slope and *pA*_2_ values (the x intercept) for each experiment (N = 6) from which mean and SEM were calculated. The Schild slope using citalopram concentrations up to 50 µM was 1.19 ± 0.09 and the *pA*_2_ was 4.79 ± 0.07. This is broadly consistent with a competitive mechanism of action with a *K_d_* = 16 µM. When CRPXL *pA*_50_ values for 100 µM citalopram were included, the Schild slope increased to 1.60 ± 0.05. This suggests that as the citalopram concentration increases, the inhibition deviates from a competitive pattern.

**Supplementary Figure S1: CRPXL-induced and U46619-induced calcium store release.** Calcium (Ca^2+^) release from intracellular stores was monitored in Fura-2-loaded platelets. Preliminary experiments (N = 1 blood donor) identified concentrations of **(a and b)** cross-linked collagen-related peptide (CRPXL) (0.5 µg mL^-1^) or **(c and d)** U46619 (0.2 µM) which induce near-maximal increases in [Ca^2+^]_cyt_. After the addition of agonist, the [Ca^2+^]_cyt_ was recorded for 3 minutes. The maximum increase in [Ca^2+^]_cyt_ following agonist addition (Max. Δ[Ca^2+^]_cyt_) was used to generate concentration-response curves, using the four-parameter logistic (4PL) model (N = 1 blood donor). R^2^ is the coefficient of determination. Red boxes indicate the agonist concentration used in subsequent experiments.

**Supplementary Figure S2: Citalopram inhibits ionomycin-induced platelet aggregation.** Ionomycin-induced platelet aggregation. **(a and b)** Preliminary experiments (N = 1 blood donor) identified the concentration of ionomycin (0.5 µM) which induced near-maximal platelet aggregation. R^2^ is the coefficient of determination. **(c)** Example traces for ionomycin-induced aggregation in platelets pre-incubated with citalopram (ionomycin = 0.5 µM, citalopram = 0, 50, 100 & 200 µM) for approximately 5 min. Arrowheads indicate time points of ionomycin addition. **(d)** The maximum extent of aggregation (Max. Aggregation) of platelets pre-incubated with a range of citalopram concentrations (0, 50, 100 & 200 µM) was used to generate concentration-response curves, using the four-parameter logistic (4PL) model, with the *Max* parameter constrained to zero: *pIC*_50_ = 3.98 ± 0.09 (N = 4 blood donors).

**Supplementary Figure S3: Citalopram is not cytotoxic to neutrophils.** Lactate dehydrogenase (LDH) release from neutrophils incubated for 10 min with various citalopram concentrations (0, 10, 20, 50, 100, 200 & 500 µM). Calcium-free Tyrode’s (CFT) or lysed neutrophils were used as negative and positive controls, respectively (N = 5 blood donors). Non-visible error bars lie within the translucent symbols. (Abs = absorbance.)

**Supplementary Figure S4: Citalopram inhibition of collagen-induced platelet activation is reversible. (a)** Diagram outlining the experimental design to test the reversibility of platelet inhibition by citalopram. Platelets were pelleted by centrifugation in the presence of prostaglandin E_1_ (PGE_1_, 1 µM) and resuspended in fresh calcium-free Tyrode’s (CFT). **(b)** *pEC*_50_ values were derived from the concentration-response curves in **(d)** and **(f)** (N = 5 blood donors). **(c and d)** Two stocks of washed platelets were either untreated (1) or treated with 100 µM citalopram (2). Samples were aliquoted to measure collagen-induced aggregation. **(e and f)** Stocks (1) and (2) were pelleted and resuspended in fresh CFT and left to rest for 1 hour. Platelet aggregation was then measured in untreated resuspended platelets (3), citalopram-treated resuspended platelets (4), and citalopram-treated resuspended platelets with a second citalopram treatment of 100 µM (5).

**Supplementary Figure S5: Citalopram inhibition of U46619-induced platelet activation is reversible. (a)** Diagram outlining the experimental design to test the reversibility of platelet inhibition by citalopram. Platelets were pelleted by centrifugation in the presence of prostaglandin E_1_ (PGE_1_, 1 µM) and resuspended in fresh calcium-free Tyrode’s (CFT). **(b)** *pEC*_50_ values were derived from the concentration-response curves in **(d)** and **(f)** (N = 4 blood donors). **(c and d)** Two stocks of washed platelets were either untreated (1) or treated with 100 µM citalopram (2). Samples were aliquoted to measure U46619-induced aggregation. **(e and f)** Stocks (1) and (2) were pelleted and resuspended in fresh CFT and left to rest for 1 hour. Platelet aggregation was then measured in untreated resuspended platelets (3), citalopram-treated resuspended platelets (4), and citalopram-treated resuspended platelets with a second citalopram treatment of 100 µM (5). The second pelleting and resuspension step has a clear effect on the responsiveness of the platelets to U46619. Nevertheless, the equivalence of the responses in conditions (3) and (4) indicate that the effect of citalopram was reversible.

******

**Supplementary Figure S6: Instantaneous platelet inhibition by citalopram.** **(a)** Representative aggregation traces for either untreated platelets, or platelets pre-incubated with 100 µM citalopram for either 30 or 60 seconds before stimulation with collagen (1 µg mL^-1^). 0 seconds represents co-administration of both citalopram (100 µM) and collagen (1 µg mL^-1^). **(b)** Following collagen addition, the maximum extent of aggregation over 6 minutes (Max. Aggregation) was quantified (N = 4 blood donors).

**Supplementary Figure S7: Uncropped images of X-ray films of Western blots showing levels of Rap1-GTP and total Rap1 in platelet lysates.** Platelets were either untreated or treated with (*RS*)-citalopram (200 µM) for approximately 5 minutes, and then either left unstimulated or stimulated with cross-linked collagen-related peptide (CRPXL, 0.5 µg mL^‑1^) or U46619 (0.2 µM). Samples from 4 blood donors were used, labelled A-D. Samples were not randomly allocated to wells. Lane 1 was used for molecular weight markers. * Indicates the blot presented in Figure 2a. The images shown here are of the X-ray films used for the quantification of Rap1-GTP reported in Figure 2a.

**Supplementary Figure S8: Uncropped images of X-ray films of Western blots showing levels of Rap1-GTP and Rap1 in neutrophil lysates.** Neutrophils were either untreated or treated with (*RS*)-citalopram (200 µM) for approximately 5 minutes, and then either left unstimulated or stimulated with platelet-activating factor (PAF, 1 µM). Samples from 4 blood donors were used, labelled E-H. Samples were not randomly allocated to wells. Lane 1 was used for molecular weight markers. * Indicates the blot presented in Figure 3c. Bands with a molecular weight of approximately 40 kDa are likely to be the GST-RalGDS-RBD fusion protein. The images shown here are of the X-ray films used for the quantification of Rap1-GTP reported in Figure 3c.

**Supplementary Figure S9: Protein sequences for recombinant CalDAG-GEFI and Rap1B.** Both CalDAG-GEFI and Rap1B were cloned from human genes into a protein expression vector p15LIC2 6xHis, which was purified in *E. coli*. CalDAG-GEFI contained a C-terminal truncation (p.(Ala552_Leu609del)). The C-terminal of Rap1B was also truncated (p.(Lys168_Leu184del)). This truncation removed disordered regions to improve stability during the purification process, while leaving all the functional domains intact. Sequences for native CalDAG-GEFI (UniProtKB-Q7LDG7) and Rap1B (UniProtKB-Q7LDG7) are provided for direct comparison.
